# Numerical Exposure Assessment Method for Low Frequency Range and Application to Wireless Power Transfer

**DOI:** 10.1371/journal.pone.0166720

**Published:** 2016-11-29

**Authors:** SangWook Park, Minhyuk Kim

**Affiliations:** 1 EMI/EMC R&D Center, Reliability & Safety R&D Division, Korea Automotive Technology Institute, Cheonan, Korea; 2 Department of Electrical and Computer Engineering, Seoul National University, Seoul, Korea; West Virginia University, UNITED STATES

## Abstract

In this paper, a numerical exposure assessment method is presented for a quasi-static analysis by the use of finite-difference time-domain (FDTD) algorithm. The proposed method is composed of scattered field FDTD method and quasi-static approximation for analyzing of the low frequency band electromagnetic problems. The proposed method provides an effective tool to compute induced electric fields in an anatomically realistic human voxel model exposed to an arbitrary non-uniform field source in the low frequency ranges. The method is verified, and excellent agreement with theoretical solutions is found for a dielectric sphere model exposed to a magnetic dipole source. The assessment method serves a practical example of the electric fields, current densities, and specific absorption rates induced in a human head and body in close proximity to a 150-kHz wireless power transfer system for cell phone charging. The results are compared to the limits recommended by the International Commission on Non-Ionizing Radiation Protection (ICNIRP) and the IEEE standard guidelines.

## Introduction

The concept of wireless power transfer (WPT) technique has been firstly proposed by Nikolas Tesla a century ago. Since a WPT technique using high resonance has been reported by MIT (Massachusetts Institute of Technology) research team in 2007 [1], many researchers have been studying on the WPT technique. Such studies were summarized in [2], and historical review of this technique was outlined as WPT has been researched applications for medical implants, induction heaters, inductive power transfer systems, and wireless charging systems for portable equipment [3–11]. Recently, WPT technique for cellular phone charging has been commercialized by the Qi standard of the Wireless Power Consortium [12]. The radiation level of electric, magnetic, and electromagnetic fields (EMFs) generated from the WPT system is extremely strong compared to that of EMFs from wireless communications. According to the specifications of the Wireless Power Consortium and AirFuel Alliance [13], charging powers are 5W to 15W for mobile devices such as cell phones and tablets, up to 100W for consumer electronics, and up to 2kW for kitchen utensils. Moreover, for the WPT applications of electric vehicles, charging powers can range from 3.3 kW to 22kW for light duty vehicles and reach 100kW for heavy duty vehicles. With continued increases in transmitted power level and transmission distance for WPT through the air, we should carefully consider electromagnetic compatibility (EMC) and the adverse health effects of EMFs exposure in close proximity to a WPT system [14].

To consider the safety issues related to human exposure to EMFs generated from the WPT system, we should evaluate internal electric fields in a human body and confirm compliance with international safety guidelines [15–18]. Due to the difficulty of measurement, numerical methods are often used for exposure assessment with anatomical human body models [19–22]. For computation of the bioelectric field, the full-wave finite-difference time-domain (FDTD) method [23] is commonly used to evaluate the internal electric field of various tissues in a human body at radio frequencies. For mid-range wireless power research, the quality factor for both transmitting and receiving resonant circuits should be large [1], and operating a WPT system at a higher resonant frequency is one of the approaches utilized to have a high quality factor. However, the operating frequencies of approximately few and/or hundreds of kHz band are discussed due to the energy efficiency and costs of overall system. Moreover, the lower operating frequency has the benefit of compliance with international guidelines because the reference levels for exposure to time-varying EMFs decrease with frequency to around 10 MHz. For operating frequency, the Wireless Power Consortium adopts a 110 kHz to 205 kHz range, and AirFuel Alliance adopts around 115 kHz, 227 kHz, and 6.78 MHz. Operating frequency bands of 20 kHz, 60 kHz, and 85 kHz are indicated for electric vehicle charging. Therefore, it is difficult to apply the conventional FDTD method to dosimetry for WPT systems in this frequency range due to the increased number of time steps. For numerical dosimetry for WPT systems, a numerical technique for a low frequency band is substantial. Various exposure scenarios should be evaluated and the fast algorithm will be helpful to process big data [24–26].

The impedance [27] and scalar-potential finite-difference (SPFD) methods [28] based on the quasi-static (QS) approximation have been used at low frequencies (frequencies up to a few megahertz). The previous works [29–32] on numerical dosimetry for approximately 10 MHz operating WPT systems have used full-wave analysis (FDTD method) and QS analysis (impedance and SPFD methods considering only incident magnetic fields) because both are available in this frequency range. As previous descriptions have indicated, a low-frequency analysis should be conducted for dosimetry for WPT systems because the operating frequency for nearly all WPT systems is in the range of a few tens of kilohertz to few hundreds of kilohertz. The QS-FDTD method has been used to compute induced currents in human models for the WPT frequency range (100 kHz to 10 MHz) [33]. However, the models were exposed to only uniform magnetic fields, in which two plane waves are superposed with different polarizations and propagation directions to cancel out electric fields, while non-uniform electric and magnetic fields are generated from a WPT system.

In this paper, a numerical exposure assessment method is proposed for low frequency range analysis. To verify our method, the internal electric field in a sphere model as a simplified human phantom model exposed to magnetic dipole is obtained by the proposed method and compared with theoretical results. Moreover, a WPT system for cell phone charging operating at 150 kHz frequency is designed, and dosimetry for the system is conducted with a human body model. Finally, we discuss the compliance of the WPT system with international safety guidelines.

## Numerical Dosimetry Method

### Theory

**Fig 1:**
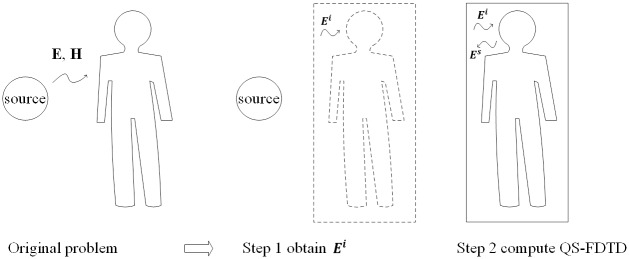
Illustration of two-step process to compute a bioelectric field problem.

The proposed numerical dosimetry method is conducted by a two-step process, similar to the previous work [31], instead of using full-wave analysis for the original problem all at once, as shown in Fig 1. The first step is to obtain the electric field generated from a source, which will be generated from a source, such as a WPT system, power line, or induction heater in the absence of a human body. The electric field can be obtained by any theoretical or numerical methods and should be located in the space occupied by a human body. In the second step, the induced electric field in a human body is calculated with a scattered-field FDTD method [23] by regarding the electric field obtained in the previous step as the incident electric field to the human body. It is noted that total electric and magnetic fields can be obtained with only the incident electric field, without the incident magnetic field in the scattered-field FDTD method. At this time, however, the QS-FDTD method is employed [34] instead of the conventional FDTD method for low-frequency analysis.

Using the QS-FDTD method, the number of time steps can be considerably decreased due to rapid convergence within a much shorter time than one full period in the quasi-static system, whereas the conventional FDTD method has to simulate several periods to reach the steady state. Under the quasi-static condition, the external fields outside a human body all have the same phase as the incident field. However, the internal fields inside a human body are proportional to the time derivative of the incident field. If a ramp function is used as an incident field, the external fields change linearly, whereas the internal fields remain constant. To avoid high-frequency contamination of the incident field, the following modified ramp function is generally used:

$$E_{inc}=\{\begin{array}{cc} 0, & -\infty <t\leq t_{0}\\ cosh\left(t-t_{0}\right), & t_{0}<t\leq \tau \\ \alpha \times \left(t-\tau \right), & t>\tau \end{array}$$ (1)

In this modified ramp function, α is the desired slope, which is related to the peak amplitude and frequency of the sinusoid wave source. The constant *t*_0_ is the starting time of the ramp excitation function, and the constant τ is generally set by hundred time steps. However, it is noted that the computation of the QS-FDTD method is simply implemented by using the same algorithm (code) as the FDTD method.

**Fig 2:**
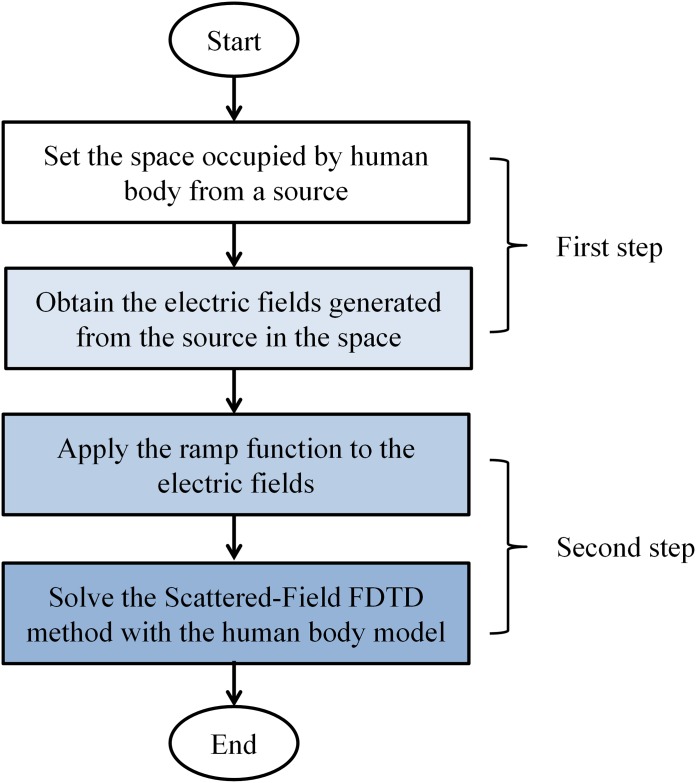
Flowchart of the proposed method.

The unique feature of the proposed method, as compared to previous research [33–35], is that the incident field can not only be uniform fields but also arbitrary non-uniform fields by using a two-step process. A key concept of this method is that the non-uniform electric fields of a system desired for exposure assessment in the first step will be used as the incident fields in the second step. To describe these non-uniform fields as the incident fields, we should consider every different magnitude of the non-uniform field. The flowchart of our approach can be expressed as in Fig 2.

### Verification

The main goal of this work is to estimate internal electric fields in a human body exposed to a WPT system at a low operating. Thus, we chose a source of magnetic dipole having EMF distribution similar to that of a WPT system. The internal electric fields in a sphere model as a simplified human phantom exposed to the magnetic dipole are calculated by the proposed method and compared to Mie theory’s calculation [36]. The relative and absolute errors are investigated to verify our method and to find out locations of maximum error.

**Fig 3:**
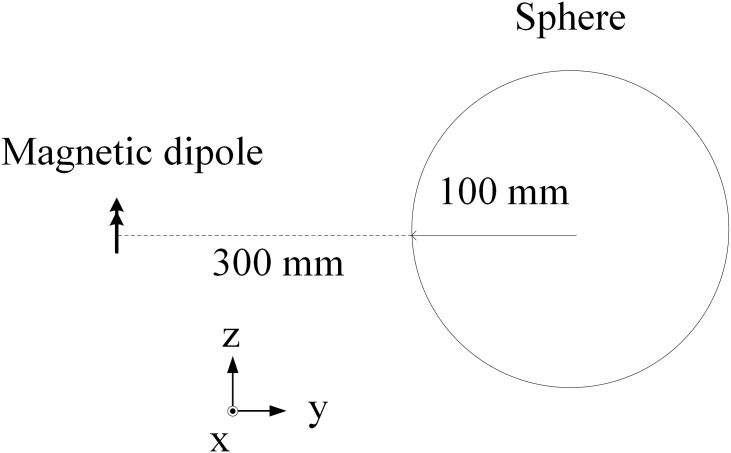
Magnetic dipole in reference to the lossy dielectric sphere.

**Fig 4:**
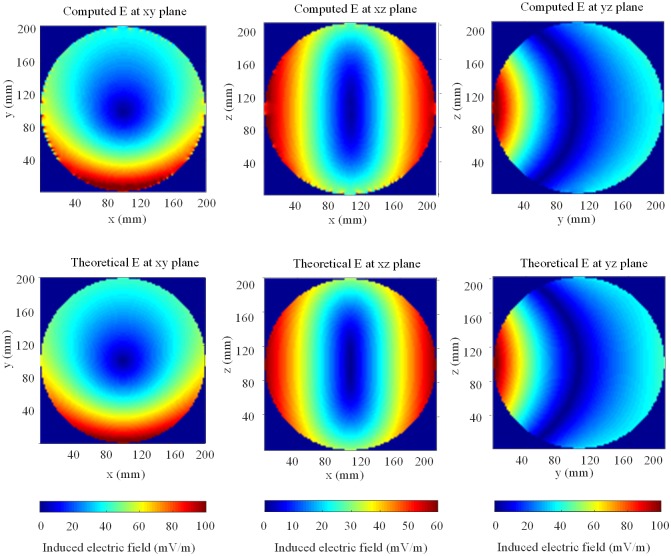
Internal electric field distribution in a sphere model exposed to a 1-MHz magnetic dipole for cross sections (computed vs theoretical). The upper and under figures show the computed and theoretical results, respectively.

Fig 3 shows the sketch of a sphere model in reference to a magnetic dipole. The distance between the center of the sphere model and the magnetic dipole is 400 mm. The radius of the sphere is 100 mm, and the magnetic dipole is in the z-polarization. The relative dielectric constant and conductivity of the sphere are 40 and 1 S/m, respectively. Through this paper, all simulations were conducted with a grid of 2 mm resolution. Convergence of this calculation was reached after 2000 time steps. Fig 4 shows the internal electric field strengths by the proposed method at cross sections in three directions, along with the corresponding theoretical solution for comparison. It is clearly seen that the field spatial distributions throughout the sphere are in good agreement with the theoretical solution.

**Fig 5:**
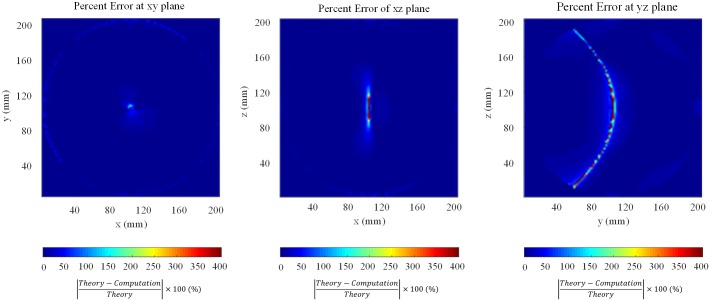
Relative error distribution at the cross sections.

Table 1 provides point-by-point relative and absolute errors for maximum and average field. Relative errors of maximum and average field are 1.72% and 0.01%, respectively. Relative errors at interior points are generally less than 1%. Fig 5 shows the relative error distributions at the cross sections. We can find the high relative errors concentrated at the surface of the sphere due to the staircase error. Relative errors range from 20% to 50% at the surface of the sphere. We can also find very large relative errors around the center related to zero field value in the theoretical solution. It is not substantial for human exposure assessment, however, because these field values are very small. The calculated values close to boundaries and zero values in the theoretical solution are overestimated, but by combining the qualitative and quantitative results, the proposed method accurately predicts the induced field distribution and field values in the interior of a lossy dielectric object such as a human body.

**Table 1:** Theoretical vs Computed electric field in a sphere model exposed to a 1-MHz magnetic dipole.

	Maximum	Average
Theoretical electric field (mV/m)	104.908	25.933
Computed electric field (mV/m)	106.709	25.935
Relative error ^a^ (%)	1.72	0.01
Absolute error ^b^ (μmV/m)	1.801	2.45e-3

^a^Difference calculated by $\left|\frac{Theory-Computation}{Theory}\right|\times 100$.

^b^Error field calculated by |*Theory* − *Computation*|.

## Human Exposure Assessment for a WPT System

In this section, dosimetry for a WPT system is conducted by using the proposed method. The size and operating frequency of the WPT system are presently designed by referring to the uniquely commercialized Qi standard. Exposure assessment for the system is computed with head and whole body of human voxel model. The results are discussed in regards to international safety guidelines.

### WPT system

**Fig 6:**
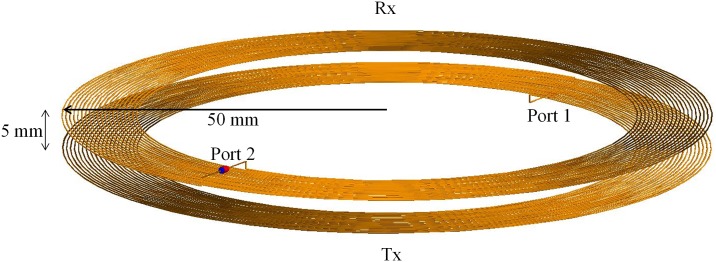
Configuration of transmitting and receiving coils for wireless power transfer.

Fig 6 shows the WPT system designed for cell phone charging. A practical WPT system uses ferrite sheet for electromagnetic interference (EMI) shielding to prevent eddy currents on it. However, in order to consider the worst exposure case, our system is simply designed without the EMI shielding. According to Qi standard, the operating frequency band ranges from 110 kHz to 205 kHz. Thus we added a lumped capacitance to the coil to operate (resonate) at 150 kHz in the middle of the frequency range. The diameter of coil was selected to be 100 mm with consideration to the size of a cell phone. Table 2 provides the parameters of the system. The transmitting coil and receiving coil are symmetrically designed, and the power transfer distance is set by 5 mm. Both ports are terminated with 50 Ω. In this condition, the power transfer efficiency is about 90%.

**Table 2:** Design parameters for a WPT system.

Operating frequency	150 kHz
Coil inductance	0.412 μH
Resonance capacitance	0.273 nF
Number of turns	20
Coil diameter	100 mm
Distance between coils	5 mm

### International safety guidelines

In the International Commission on Non-Ionizing Radiation Protection (ICNIRP) and the IEEE standard guidelines up to 300 GHz, established adverse health effects depend on the frequency and intensity of the EMF. The electrostimulation effects dominate at low frequency, and the thermal effects dominate at high frequency. Thus, in the intermediate frequency region, we have to consider both effects. To protect against both effects, the ICNIRP and the IEEE standard recommend limits in the region from 100 kHz to 10 MHz and in the region from 100 kHz to 5 MHz, respectively. It is noted that in 2010, new ICNIRP guidelines (1 Hz to 100 kHz) were published to replace the low-frequency part of the 1998 ICNIRP guidelines (up to 300 GHz). The 99th percentile value of the internal electric field for a specific tissue (E_99_), the induced current density (J)—which is used in ICNIRP guidelines—and the average value of the electric fields over a 5-mm-long straight-line segment oriented in any direction within the tissue (E_5mm_)—which is used in the IEEE standard—are the metrics for human exposure limits to protect against electrostimulation effects. The specific absorption rate (SAR) is used as the metric for human exposure limits to protect against thermal effects in both guidelines. These metrics are called basic restrictions, which refer to restrictions on exposure to time-varying EMFs based directly on established health effects. Table 3 provides the basic restriction at 150 kHz of the WPT operating frequency.

**Table 3:** Metrics of ICNIRP and IEEE guidelines at 150 kHz.

Metrics		Values	Guidelines
J (A/m^2^)		0.3	ICNIRP1998
E_99_ (V/m)		20.25	ICNIRP2010
E_5mm_ (V/m)	brain	44.175	IEEE
	heart	847.006	
	limbs	94.0299	
	other tissues	31.3881	
SAR_10g_ (W/kg)	head and trunk	2	ICNIRP/IEEE
	Limbs (pinnae ^a^)	4	
SAR_wm_ (W/kg)		0.08	

SAR_10g_ is the localized SAR average of any 10g cubical volume of tissue.

SAR_wm_ is the whole-mass average SAR.

^a^IEEE includes pinnae together with limbs for localized SAR.

### Human model and exposure conditions

A whole-body voxel human model is used for dosimetry for a WPT system. The heterogeneous model of the human body TARO [20] is based on the accumulated magnetic resonant imaging (MRI) data of adult Japanese volunteers. This anatomically realistic Japanese adult male model possesses 2-mm spatial resolution and 51 tissues and organs. In all computations, the x-axis of the coordinate system is from left to right, the y-axis is from back to front, and the z-axis is from foot to head as 320×160×866 voxels. The various tissues and organs were assigned from Gabriel’s Cole-Cole models [37].

**Fig 7:**
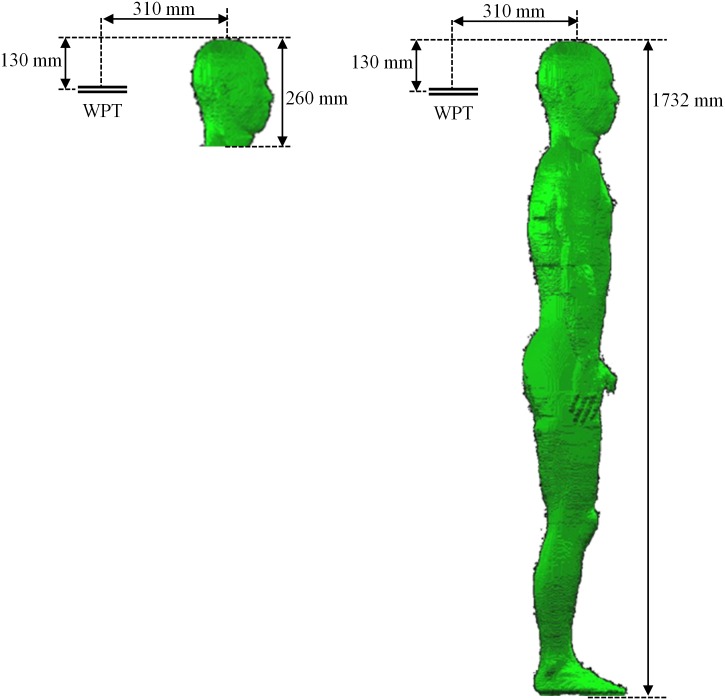
Position of the human body model in reference to a WPT system.

Two exposure conditions are computed as illustrated in Fig 7. The head and whole body of TARO model are situated in front of the WPT system designed in this paper. The distance between the two centers of the WPT and TARO is 310 mm. Only the head model and the head of the whole-body model are located at the exact same position. All induced quantities were computed for an input power of 1 W.

### Dosimetry results and discussion

The incident electric fields generated from the WPT system are computed by using the method of moment (MoM) [38]. The internal electric field, induced current density, and SAR are evaluated based on the proposed method. Steady state for the head and whole-body models were reached after 2000 and 3000 time steps, respectively. In the conventional FDTD method, the duration of one time step would be $\Delta \text{t}=\Delta \text{x}/\sqrt{3}c=0.38\;ps$ with spatial discretization of Δx = Δy = Δz = 0.2 mm at 150 kHz from the Courant stability criterion. Generally, the conventional FDTD method requires a few periods of the source field. The required number of the time steps for four periods would be approximately 1.7×10^7^. In this frequency, the proposed method reduced the simulation time by nearly 5773-fold compared to the conventional FDTD method. However, the required memory for the proposed method is the same as that of the conventional FDTD method because they use the same algorithm.

**Fig 8:**
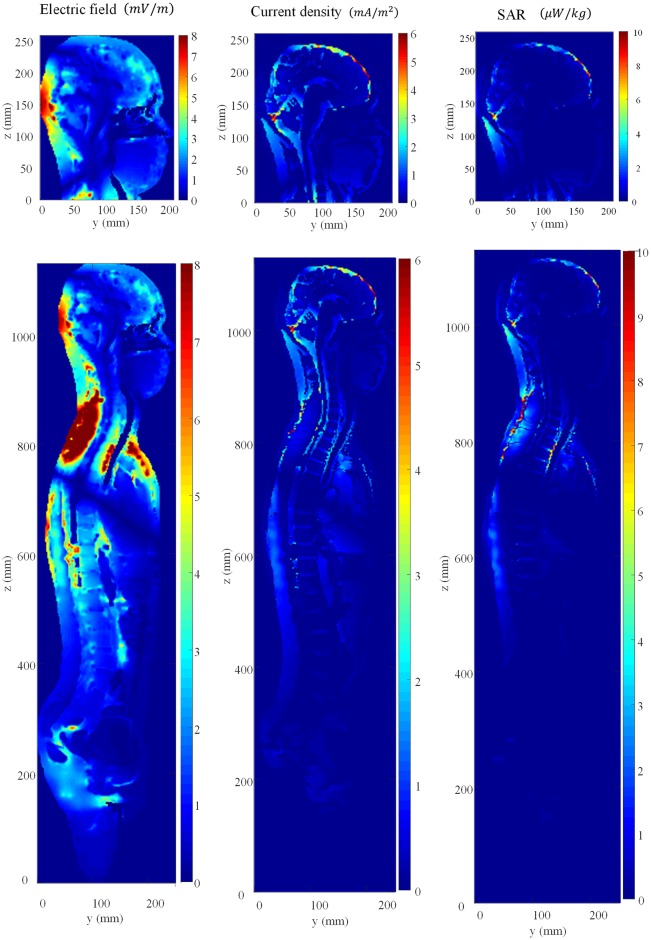
Distributions of induced electric field, current density, and SAR in head and whole-body models in the cross section at xy plane, respectively.

**Fig 9:**
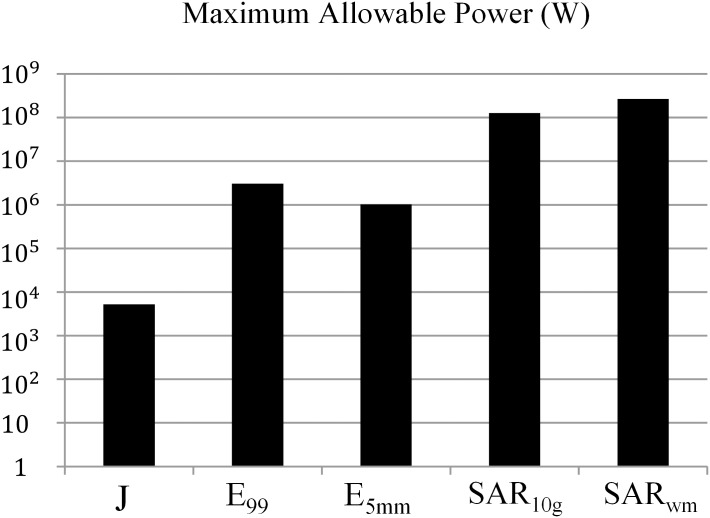
Maximum allowable power to satisfy the basic restrictions.

Fig 8 shows the electric field, current density, and SAR distributions for the head and whole-body models. The two distributions for the head and the whole body are slightly different. Table 4 provides the induced quantities for the head and whole body. We calculated not only the metrics suggested by the international guidelines but also the induced quantities in the central nervous system (CNS)—which is important for the electrostimulation effects—and the localized SAR average of any 1 g cubical volume of tissue (SAR_1g_). The results show that the induced quantities in the whole body are larger than those in the head only except for the SAR_wm_ due to the larger circular conductive loop in the whole body compared to that in the head. On the other hand, the induced quantities in the central nervous system for the head and the whole body are both similar to each other. Therefore, under these computation conditions, the results suggest that the induced quantities of CNS can be computed by using the head model instead of the whole-body model, whereas the other induced quantities should be computed by using the whole-body model. We investigated the maximum allowable powers (MAP) to comply with the basic restrictions suggested by each safety guideline as shown in Fig 9. In this computation condition, the results show that the basic restrictions to protect from electrostimulation effects (J, E_99_, E_5mm_) are more severe than the basic restrictions to protect from the thermal effects (SAR) for all guidelines, while for the current density (J), the limits recommended by the 1998 ICNIRP are the most severe. Thus, in order to comply with the human exposure regulations for the WPT system operating at 150 kHz frequency, we should first consider the basic restrictions to protect from the effects of electrostimulation.

**Table 4:** Induced quantities in head and whole body.

	Head (tissue name)	Whole body (tissue name)
J (mA/m^2^)	3.48 (CSF)	4.15 (Muscle)
J_cns_ (mA/m^2^)	1.40 (Grey matter)	1.41 (Cerebellum)
E_99_ (mV/m)	9.36 (Cortical bone)	11.57 (Cortical bone)
E_99cns_ (mV/m)	7.61 (CSF)	7.63 (CSF)
E_5mm_ (mV/m)	19.53 (Cortical bone)	31.09 (Fat)
E_5mmcns_ (mV/m)	10.93 (Grey matter)	10.95 (Grey matter)
SAR_1g_ (nW/kg)	6.97 (CSF)	21.86 (Muscle)
SAR_10g_ (nW/kg)	2.63 (Cerebellum)	15.65 (Muscle)
SAR_wm_ (nW/kg)	0.44	0.30

CSF is the abbreviation for Cerebrospinal fluid.

The induced quantities including the cns subscript represent the values in the central nervous system

## Conclusions

A two-step approach using the quasi-static FDTD has been provided to compute human exposure assessment for an arbitrary non-uniform field of low frequency range. A verification of our method against a theoretical solution for a lossy sphere as a simplified human phantom in the proximity of a magnetic dipole source attests to its accuracy. The result suggests that the proposed method provides an effective tool for computations of induced electric fields in the human body in close proximity to any sources. For application, induced electric field, current density, and SAR in the head and whole body of a human in the proximity to a WPT system for cell phone charging operating at 150 kHz frequency have been computed with the proposed method. The results showed that the limits of induced electric field and current density should be carefully considered in comparison to SAR limits. The proposed method will be applied for accurate evaluation of fields in the body in various EMF environments, such as household, industry, automobile, and medical uses. For future work, the dosimetry for electric vehicle wireless chargers requiring high power will be conducted.
